# The formidable guardian: Type 3 immunity in the intestine of pigs

**DOI:** 10.1080/21505594.2024.2424325

**Published:** 2024-11-04

**Authors:** Zhipeng Yang, Dou Zhang, Zhoudan Jiang, Jian Peng, Hongkui Wei

**Affiliations:** a Department of Animal Nutrition and Feed Science, College of Animal Science and Technology, Huazhong Agricultural University, Wuhan, China; b The Cooperative Innovation Center for Sustainable Pig Production, Wuhan, China; c Frontiers Science Center for Animal Breeding and Sustainable Production, Wuhan, China

**Keywords:** RORγt, Th17, γδT17, ILC3, infection, nutrients

## Abstract

Well-intestinal health is crucial for better growth performance in pigs. Type 3 immunity, which is one of the three types of immune responses in mammals, plays a vital role in maintaining intestinal homoeostasis. Therefore, we initially introduce the type 3 immune cells in the intestine of pigs, including their distribution, development, and function. We then discuss the type 3 immune response under infection, encompassing bacterial, fungal, and viral infections. It also covers two major stresses in pigs: heat stress and weaning stress. Lastly, we discuss the effects of various nutrients and feed additives on the regulation of the type 3 immune response in pigs under infection. This review aims to contribute to the understanding of the interaction between infection and type 3 immunity in pigs and to illustrate how various nutrients modulate the type 3 immune response in pigs under diverse infections.

## Introduction

Maintaining intestinal immune homoeostasis is beneficial for the growth performance of pigs under various microbial infections and stress [1]. Furthermore, the pig model is an excellent animal model for the study of immunology because they are similar with humans in the genome, anatomy, and physiology [2]. Type 3 immunity involves both innate and adaptive immune responses, characterized by the expression of genes encoding interleukin-17A (IL-17A), IL-17F, and IL-22, as well as the key transcription factor retinoic acid-related orphan receptor (RORγt) [3,4]. The cells responsible for type 3 immunity are diverse, mainly including group 3 innate lymphoid cell (ILC3), gamma delta T 17 cell (γδT17), and T helper 17 cells (Th17). The research found that type 3 immunity serves a critical role in defending microbial infection, maintaining intestinal epithelial homoeostasis, and repairing intestinal epithelial injury under stress.

Hence, we discuss the spread and development of type 3 immune cells in the intestine of pigs, and the function of type 3 immune factors in the intestine, as well as discussing the type 3 immune response under infection and stress. Last, we research what and how various nutrients and feed additives regulate type 3 immune response in pigs. The study aims to contribute to the understanding of type 3 immunity in pigs, as well as to suggest a promising target for promoting intestinal health in pigs.

## Type 3 immune cells

### γδT17

T lymphocytes can be divided into two populations, αβ T cells and γδ T cells, based on their expression of different forms of the T cell receptor (TCR). As an innate subset of T lymphocytes, γδ T cells play an important role in the early immune responses against a variety of pathogens, including bacteria [5] and viruses [6], maintain mucosal tolerance [7], mediates the efficacy of tumour immunotherapy [8]. γδT17 cells belong to the type 3 innate immune cells, which are characterized by IL-17A-secreting, commonly found in the mucosal surfaces and secondary lymphoid organs such as the intestine and skin [9].

Pigs are considered to have a higher abundance of γδ T cell species compared with rodents and humans [10]. A study found that rodents and humans had a minor population of γδ T cells (1–5%) in their blood lymphocyte pool, whereas pigs had a major percentage (15–60%) of γδ T cells in their blood lymphocyte pool, indicating γδ T cells play an important role in the immune system of pig [11]. The absolute number of pig γδ T cells in the blood increased gradually during 1–3 weeks and increased rapidly after weaning and by 19–25 weeks, γδ T cells account for more than 50% of circulating lymphocytes [12]. Further, the low birth weight piglets showed a lower percentage of γδ T cells in peripheral blood mononuclear cell (PBMC), highlighting their importance in the early development and protection of the immune system [13]. In the mesenteric lymph nodes (MLNs) of pigs, the total γδ T cells did not change from 1 day to 6 months of age [14], while the ratio of CD8^+^γδ T cells and γδT17 cells significantly increased from 0 days of age to 6 months of age [15], which indicate that the development of γδT17 in the MLNs of pigs is age-dependent. However, the change of γδ T cells in the early life of the mice model is distinct from pig. The γδ T cells in the small intestine and MLNs of neonates of mice were double fold of adults. In the jejunum of human, γδ T cell frequencies are initially high in early life but decrease between ages 1 and 5 to levels seen in adults. Conversely, γδ T cell frequencies in the blood rise from birth to 12 years, despite being low in adults [16].

Moreover, the IL-17A secreting ability of γδ T cells in the neonates was also stronger than that of adults of mice, which promoted the host defence against *Clostridium difficile* infection [17]. It is well known that γδ T cells produce IL-17A, one of the most important master regulators, shapes the immune response by orchestrating downstream cytokine and chemokine production by other cells [18]. However, the IL-22-producing γδ T cells identified in mice and humans are not found in the pig [19–21]. Moreover, in other farm animals such as cattle, γδ T cells were one of the main producers of IL-22 [22].

### Th17

Th cells were also called CD4^+^T cells, because of the characteristic of the surface receptor CD4 protein. CD4^+^ T cells play a well-established role in the protection of microbial infections including bacteria [23], viruses [24], parasites [25], and the progress of experimental colitis [26]. CD4^+^T cells can be divided into various subsets including Th1, Th2, and Th17 according to the characteristics of cytokines, and Th17 was defined as CD4^+^IL-17A^+^T cell [27]. RORγt^+^ regulatory T cell (Treg) was found in 2014 and has been researched widely recently, can suppress inflammatory reactions, and prevent overwhelming immune responses, thereby maintaining the delicate balance of the intestinal immune system [28,29]. In mice, approximately 10 to 25 percent of the peripheral RORγt^+^ Treg cells showed the expression of IL-17A, while the colonic RORγt^+^ Treg cells did not generate IL-17A [30].

In pigs, CD4^+^T cells, CD3^+^T cells, and CD3^+^CD4^+^ Th cells in the blood significantly increased from born to 7 weeks of age, especially in the first week of life and the weaning period, while staying at a steady level during 7 weeks to 25 weeks of life [12]. CD4^+^ Th cell was identified as one of the main sources of IL-17A in the blood of the pig [18]. The ratio of the Th17 cells in the MLNs gradually increased during the suckling period of the pig, and at 6 months of age, the pig had a double fold of Th17 cells compared with 28 days of age, which showed that the development of Th17 occurs both in the suckling and weaning period [15]. In adult humans, there are about 7% of Th17 in the small intestine and the frequency of Th17 in the small intestine is higher than PBMC [31]. However, RORγt^+^ Treg has not been identified in pigs yet but was identified in human [32].

### ILC3

Innate lymphoid cells (ILCs) were a novel cluster of immune cells found in 2008 in mice [33] and were found to play multiple roles in maintaining intestine homoeostasis recently, for example, infection defence [34], immune tolerance [35] and tissue repair [36]. ILCs can be divided into four main subsets including ILC1, ILC2, ILC3, and ILCreg. Among them, the ILC3 was dependent on the transcription factor RORγt and characterized by producing IL-17 and IL-22. ILC3 plays a crucial role in type 3 immunity, contributing to intestinal regeneration, bacterial infection responses [37], and immune tolerance [34]. Specifically, ILC3 is involved in modulating the intestinal barrier function and promoting the secretion of antimicrobial peptides, thereby aiding in the defence against extracellular pathogens [38]. In addition, ILC3 is essential for directing the development of intestinal macrophages and maintaining mucosal homoeostasis, making them key players in intestinal immunity and inflammation [39].

With the development of single-cell RNA sequencing (scRNA-seq), the intestinal ILCs family of pigs was identified in 2022 and 2023, respectively, [40,41]. ScRNA-seq showed that the intestinal ILCs highly expressed *CD2* and lacked *CD3E*, *CD79A*, and *CD172α*, which belong to T, B, and myeloid lineage leukocyte markers, respectively. Interestingly, in lamina propria lymphocytes (LPLs) of the jejunum, ILC3 were the most abundant Lin^−^CD45^+^ cells. Meanwhile, scRNA-seq results showed that ILC3 highly expressed *IL-23 R*, *aryl hydrocarbon receptor* (*AHR)*, *IL-7 Rα*, *RORC*, and *IL-22* compared to other types of immune cells, which is similar to human [40,41]. Fluorescence in situ hybridization (FISH) results showed that abundant *IL-22*^+^ cells lacked *CD3ε* expression, implying that most of the IL-22-expressing cells in the LPLs of pig may be the ILC3 [40]. Therefore, the researcher identified ILC3 as *IL-22*^+^*CD3ε*^−^ by FISH in the intestine of pig [40]. It is regrettable that we still do not know the pattern of developmental changes, and comparisons between the different sites of the pig intestine.

## Type 3 immune factors

### IL-17A and IL-17F

IL-17 was first confirmed in 1995 and belongs to the IL-17 family which consists of six members IL-17A-F [42]. In pigs, CD4^+^ and γδTCR^+^ T lymphocytes were sources of IL-17A [18]. While, whether IL-17A^+^ILC3 exerts in the intestine of pigs is still unknown. IL-17A plays a crucial role in regulating the host’s immune response against pathogens, which induces proinflammatory cytokines and chemokines that participate in the recruitment of neutrophils and macrophages to the site of injury. [43]. Further, IL-17A directly regulated the antimicrobial functions of paneth cells, especially the production of α-defensin, and thus maintained a microbiota homoeostatic state [44]. However, during colonic inflammation, IL-17F promoted colonic expression of the antimicrobial peptides including angiogenin 4 and phospholipase A2, and then restricted the growth of the beneficial bacteria *Clostridium bolteae* and *Clostridium clostridioforme* [45].

### IL-22

IL-22 was first confirmed in 2000 and belongs to the IL-10 family [46]. Both CD3^+^T cells and ILCs can produce IL-22, while most IL-22 is produced by ILC3 in pigs [40]. Intestinal type 3 immune cells generate IL-22, which plays a crucial role in maintaining the intestinal mucosal barrier function, particularly the epithelial layer. IL-22 promoted intestinal stem cell DNA repair, apoptosis, and epithelial regeneration [47,48]. Further, IL-22 mediated the development and function of paneth cells in mice [49], and deficient IL-22 receptors in the intestine reduced the expression of lysozyme and regenerating family member 3 gamma (Reg3γ) [50]. During weaning in mice, the increase of IL-22 promoted the differentiation of nonproliferating intestinal epithelial progenitors into mucine17 (MUC17)-expressing intestinal epithelial cells (IECs) to maintain the homoeostasis of the intestine [51]. Under the bacterial pathogen infection, IL-22 induced diarrhoea and clears intestinal pathogens by upregulating epithelial claudin-2 [52].

Compared with IL-17, IL-22 was widely researched in the pig model. Purified IL-22 reversed apoptosis and repaired the intestinal injury induced by deoxynivalenol (DON). Further, when exposed to enterotoxigenic escherichia coli (ETEC) K88 infection, IL-22 stimulated epithelial cells to release β-defensin 1, and it alleviated apoptosis induced by the infection [53]. Besides, as demonstrated by the IL-22 inhibitory effect on several intestinal diarrhoea viruses in the intestinal porcine epithelial cell line J2 (IPEC-J2) cells, including alpha coronavirus, porcine epidemic diarrhoea virus (PEDV), transmissible gastroenteritis virus, and pig rotavirus [54].

### Type 3 immune response under infection

**Figure 1.**
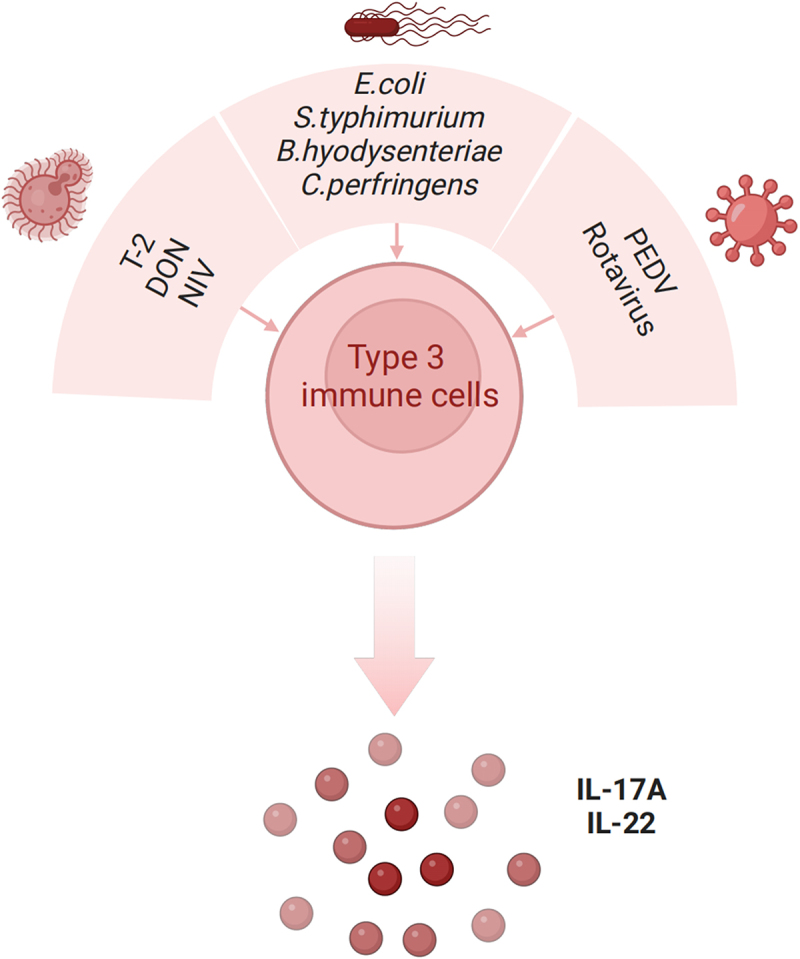
Type 3 immune response under infection. Microbial infections including viruses (PEDV and rotavirus), bacteria (*E. coli*, *S*. typhimurium, *B. hyodysenteriae*, *C. perfringens* type C), and mycotoxins (T2, DON) activate type 3 immune cells to produce cytokines IL-17 and IL-22. Created with BioRender.com.

The intestine is often susceptible to invasion by various microbes due to its exposure to the external environment. That is the reason why the intestine is the biggest immune organ in mammals, which presents about 70% of immune cells in the host [55]. Numerous studies reported that type 3 immune response served an essential role in the defence against microbial infections such as viruses, bacteria, and fungi in mice [56]. Therefore, a clearer about how the pathogen interacts with type 3 immunity in pigs will help us to alleviate the microbial infection with an efficient approach. The effect of detailed infected factors on the type 3 immune response is described in Figure 1

## Bacteria

Pathogenic bacteria are first recognized by macrophages and dendritic cells through pattern recognition receptors, which leads to the production of type 3 inducer cytokines such as IL-1β and IL-23. These cytokines then induce the phosphorylation of STAT3, which subsequently activates RORγt [57,58]. ETEC was one of the reasons induced diarrhoea in the neonatal and weaned piglet [59]. ETEC attached to the small intestine by the fimbriae, then secreted enterotoxins including heat-labile toxins and heat-stable toxins, and further weakens the intestinal barrier thus inducing intestinal inflammation [59]. Previous studies predominantly focused on the response of IECs to ETEC invasion, often overlooking the role of other immune cells.

Recent research has revealed that ETEC infection upregulates the mRNA levels of type 3 immune factors such as *IL-17A*, *IL-17F*, *IL-22*, and *RORC* in the PBMC and intestine of pigs [60–63]. To more focus, the immunofluorescence results have shown the IL-17A^+^CD3^+^T cells (Th17 and γδT17) were abundant in the jejunum of pig [60]. Additionally, the ETEC infection elevated the expression of RORγt in both the jejunum and ileum [61]. Besides, flow cytometry results indicated an increase in γδ T cells in the MLNs and spleen during ETEC infection [64]. However, the specific cytokines expressed by these γδ T cells and their precise role in ETEC infection remain unclear. Furthermore, the extent of changes in ILC3 cells in ETEC-infected pigs is still unknown.

*Brachyspira hyodysenteriae* (*B.hyodysenteriae*), another pathogen, caused diarrhoea in pigs post-weaning and during the fattening phase [65]. In the *B. hyodysenteriae* infection model, IL-17A-dominated type 3 immunity has been observed. Techniques including quantitative PCR, immunohistochemistry, and RNA ISH have all targeted IL-17A in the colon highlighting its crucial role in *B. hyodysenteriae* infection [66,67]. Despite this, the source of IL-17A is still unknown, although increases in Th and γδ T cells have been detected in the PBMC and intestines of infected pigs [68,69].

*Clostridium perfringens* (*C. perfringens*) type C infection induced severe and fatal necrotic enteritis in newborn piglets [70]. Evidence suggested that the IL-17 signalling pathway is enriched in the spleen of pigs infected with *C. perfringens* type C [71]. Additionally, Th17 cells were elevated in the gut and MLNs of pigs infected with *Salmonella* Typhimurium, another common bacterial pathogen in pigs [72].

In conclusion, type 3 immune responses, particularly involving IL-17 and IL-22, play crucial roles in defending against various bacterial pathogens in pigs, highlighting the need for further research to fully understand their mechanisms and contributions to intestinal health.

## Mycotoxins

Feed ingredients including corn, oats, barley, wheat, rye, and sorghum usually be infected by the fungi of the genera *Fusarium*, *Myrothecium*, *Spicellum*, *Stachybotrys*, *Cephalosporium*, and *Trichothecium* [73] which produce mycotoxins such as *T*-2, DON, and Nivalenol (NIV). Most mycotoxins in feed enter the host through the intestines and weaken the intestinal immune system. It has been demonstrated that mycotoxins induced inflammation, ulcers, and necrotic in the intestines of pigs [74]. However, a detailed understanding of the influence of mycotoxins on the type 3 immune response is still lacking.

In vitro experiments indicated that mycotoxins can affect the expression of transcription factors and the production of cytokines in T cells of pigs. Specifically, DON has been shown to reduce the proliferation of CD4^+^ cells in PBMC [75,76]. Additionally, DON upregulated the expression of transcription factors including T-box transcription factor 21 (T-bet) and GATA binding protein 3 (Gata3) [75]. However, there was no study on the change of type 3 transcription factor RORγt under DON treatment. Evidence showed that DON restrained type 3 cytokines expression, with *IL-17A* mRNA expression decreased in the DON-treated T cells [77]. Furthermore, the proportion of IL-17A^+^CD4^+^ and IL-17A^+^γδ T^+^ cells did change in the DON-treated PBMC [75].

Other in vitro models, such as intestinal explants can also be used to study the immune regulation of mycotoxins in pigs [78]. The mRNA and protein expression of IL-17A and IL-22 were elevated in the DON-treated intestinal explants [79–81]. Both *T*-2 and DON mycotoxins have been demonstrated the capability to influence the type 3 immune response in vivo. For example, the proportion of γδ T cells and the IL-17A level in the ileum were increased in the pigs infected with *T*-2 [82]. Exposure to DON through maternal exposure upregulated γδ T cells in neonates, while decreasing Th cells and IL-17A levels in the ileum [83].

These results indicate that mycotoxins may not directly influence type 3 immune immunity, but other cells may participate in inducing type 3 immune response in vivo in pigs. For example, exposure to DON induced the overexpression of cytokines such as IL-1β in the IPEC-J2 [84] or induced apoptosis in the pig alveolar macrophage [85], which has been reported to lead to type 3 immune response in mice.

## Virus

Though researchers typically recognized that the type 1 immune response mediated by interferon (IFN)-γ plays a crucial role in defending against viruses in pigs [86] and mice [87], recent findings highlighted the significance of type 3 immunity. IL-22, a key cytokine in type 3 immunity, has been shown to induce the proliferation and migration of IECs to promote the extrusion of rotavirus-infected IECs into the intestinal lumen of mice [88]. Moreover, IL-22 produced by ILC3 cells aids in repairing damage and protecting mice from intestinal viral infections [89].

PEDV, a classic gastrointestinal coronavirus, causes severe diarrhoea in piglets [90]. Studies have demonstrated that the PEDV infection induces the proliferation of Th cells and γδ T cells in vitro [91]. Additionally, the PEDV-infected pigs showed elevated levels of IL-17A and IL-22 in their serum [92]. Sc-RNAseq of PEDV-infected porcine small intestines has revealed that IL-22 is predominantly expressed by Th17 cells, with its expression significantly upregulated during viral infection [91]. The transwell model of immune cell migration and siRNA technology has shown that IEC-induced C-X-C motif chemokine ligand 13 (CXCL-13) activates T cell migration, indicating that the underlying mechanism of type 3 immune response may involve the secretion of cytokines and chemokines by IECs [91].

In another model, diarrhoea caused by rotavirus, which was a highly contagious viral pathogen that commonly infected epithelial cells of the small intestine worldwide and caused diarrhoea in neonatal piglets and young children [93,94]. There was an upregulation of γδ T cells in the ileum and blood, particularly the CD2^+^CD8^+^γδ T cells [95]. Similarly, findings show that AHR and IL-22 expression was elevated in the ileum during the influenza A virus infection, and the metabolism of tryptophan metabolism of gut microbes may help this phenomenon [96].

In summary, type 3 immunity, particularly involving IL-22, is crucial for defending against gastrointestinal viral infections in pigs by promoting epithelial cell responses. This highlights the importance of type 3 immunity in maintaining intestinal health and combating viral pathogens in pigs.

## Type 3 immune response under stress

### Heat stress

Pigs are not good at regulating body temperature through skin evaporation and heat dissipation because of the thick subcutaneous fat and underdeveloped juice glands, thus the pig is sensitive to heat environment [97]. The gut is one of the organs most affected by heat, a decrease in tight junctions was associated with an increase in intestinal permeability and an increase in myosin light chain kinase [98]. Next, the immune response was caused and aggravated by the translocation of luminal antigens, endotoxins, and pathogenic bacteria [99].

It has been demonstrated that the CD3^+^T cells and CD3^+^CD4^+^ Th cells were upregulated under heat stress in the ileal peyer patch of pig [100]. Moreover, RNA-seq results of the colonic mucosal showed that the Th17 cell differentiation signalling pathway enriched in the pig of heat stress [101]. Further, the *IL-17A* and *RORC* expression in MLNs and LPLs of colin was elevated by heat stress [102]. Though IL-17A mediated by type 3 immunity is induced by heat stress because of the transfer of pathogenic bacteria from the lumen, it seems over-activated and contributes to the damage of the intestine. In mice models, IL-17A administration exacerbated heat stress in a mouse model, while anti-IL-17A treatment effectively inhibited the inflammatory response [103]. Regrettably, we did not find any reports of other type 3 immunity factors in the heat stress, if IL-22 serves a repair role in the heat-induced damage is still unknown. Whereas, other research demonstrated intestinal ILC3 secreting IL-22 contributing to promoting heat production of mice under intermittent fasting [104]. Thus, it is worth investigating how other types 3 immune cells and factors affect the progress of heat stress.

### Weaning stress

Weaning was an essential stage in the life of mammals, changes in diet and social environment led to severe intestinal barrier injury including the physical barrier, the chemical barrier, and the immune barrier in pigs [105]. Moreover, early weaning (15 to 21-day weaning age) usually occurs in the production for more profit, thus the immune homoeostasis receives shock more seriously than usual.

The mRNA expression of *IL-17A* in the in the Th17 from MLNs elevated at 7 days after weaning and then decreased gradually [106]. IL-17A has usually been seen as an inflammation marker during weaning due to the over-differentiation of Th17. However, this phenomenon seems to be helping to train the protective intestinal immune response in the latter life. For example, pre-weaned mice failed to trigger a protective IL-17A response to *Giardia muris* infection, in contrast, IL-17A was upregulated after weaning [107]. The mRNA expression of *IL-22* in the jejunum of piglets gradually increased during weaning [108]. In weaning mice, neonatal progenitor enterocytes are reprogrammed into MUC17-expressing enterocytes triggered by the high level of IL-22 [51]. Indeed, a previous study demonstrated that IL-22 upregulated the mRNA expression of intestinal epithelium maturation makers such as *Arg2* and *Ada* in pig intestinal organoids [109]. The result suggested that IL-22 secreted by type 3 immune cells may repair intestinal injury, especially intestinal stem cell damage, which boosted the translation from sucking to youth in piglets.

### Nutritional interventions

**Figure 2.**
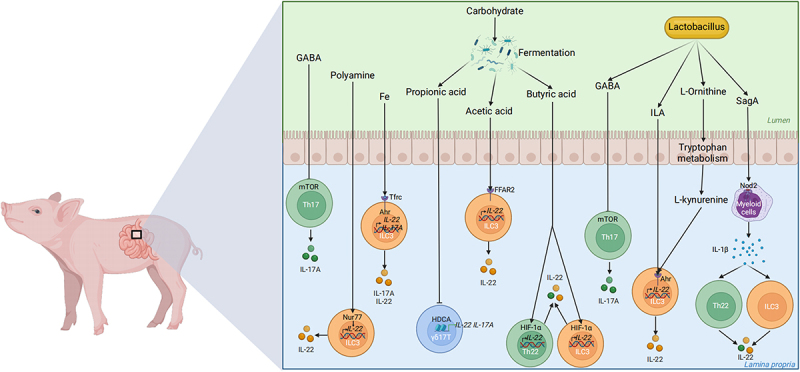
Potential mechanisms of nutritional intervention on type 3 immune cells. The complex interactions in the pig gut involve probiotics (e.g. *Lactobacillus*), nutrients (e.g. GABA, polyamine, Fe, carbohydrate) and immune cells. Carbohydrate fermentation produces short-chain fatty acids (propionic acid, acetic acid, butyric acid) influencing various immune cells (Th17, ILC3, γδT17 cells) through receptors (e.g. mTOR, FFAR2). *Lactobacillus* influences type 3 immune cells through GABA, ILA, L-Ornithine, and SagA leading to the secretion of IL-17A and IL-22, thereby enhancing gut immune response and barrier function. Created with BioRender.com.

**Figure 3.**
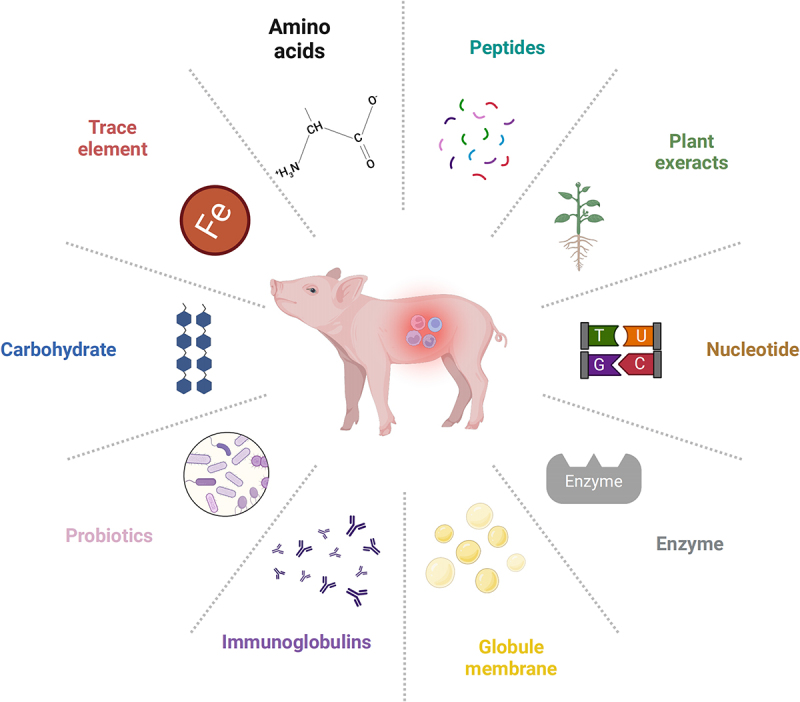
Different strategies of nutritional intervention on type 3 immune response. Key nutritional factors influencing pig gut health, including amino acids, peptides, plant extracts, nucleotides, enzymes, globule membranes, immunoglobulins, probiotics, carbohydrates, and trace elements. These components collectively affect type 3 immune response. Created with BioRender.com.

Numerous studies found that the infection or stress of pigs can be alleviated by regulating type 3 immune response through nutritional interventions. Nutritional interventions such as amino acids, peptides, carbohydrates, and trace elements, can all induce type 3 immune response (Figure 2,3), as detailed in Table 1.

**Table 1.** Effects of nutritional interventions on type 3 immune response in pigs.

Object	Model	Dose	Function	Ref.
**Amino acids**				
GABA	ETEC-infected Weaning Piglets	40 mg kg^−1^ basal diet for 21 d	Increase concentration of IL-17A in jejunum	[110]
GABA	Weaning piglets	20 mg kg^−1^ basal diet for 21 d	Inhibit the mRNA expressions of *IL-22*	[111]
Glutamate	LPS challenged piglets	1% glutamate in diet for 21 d	Decrease the mRNA expression of *RORC* and *STAT3* Decrease the mRNA expression of IL-17A	[112]
Glutamine	LPS challenged piglets	1% glutamate in diet for 30 d	Increase serum IL-17A content Increase the mRNA expression of *IL-17A*	[113]
**Trace element**				
Iron dextran Ferrous glycine	Newborn piglets	Oral administration at 25 mg/d on days 2, 7, 12, and 17	Increase protein expression of IL-22 in the colon	[114]
ZnO nano-ZnO	Weaning piglets	200 ppm/kg nano-ZnO or 2500 ppm/kg ZnO for 14 d	Nano-ZnO group had higher numbers of RORγt^+^ T cells in the MLNs.	[115]
Selenium deficiency	Weaning piglets	Selenium Deficiency for 35 d	Increase the protein and mRNA levels of IL-17A in intestine	[116]
**Peptides**				
Hen egg lysozyme	DSS induced colitis piglets	150 mg/kg BW for 5 d	Reduce the mRNA expression of *IL-17A* in the colon	[117]
Soy-derived di- and tripeptides	DSS induced colitis piglets	250 mg/kg BW for 5 d	Decrease mRNA expression of *RORC* and *IL-17A* in the colon Decrease mRNA expression of *IL-17A* in the ileum	[118]
Sodium caseinate hydrolysate	Weaning piglets	0.25 g/kg basal diet for 12 d	Downregulate mRNA expression of *IL-17A* in the colon	[119]
Casein hydrolysate	Finishing pig	4.5% of basal diet for 28 d	Decrease protein expression levels of IL-17A	[120]
Cathelicidin-BF	Weanling piglets	Intraperitoneally inject 0.6 mg/kg BW for 7 d	Decrease protein level of IL-22 in the serum	[121]
**Probiotics**				
*Lactobacillus rhamnosus*	DON-treated jejunal explants	Medium with 10^9^ CFU/mL	Reduce the mRNA expression of *IL-22*	[122]
*Bacillus cereus* *var. Toyoi*	*Salmonella*-infected weaning piglets	8.7 × 10^8^ CFU/Kg diet for 10 d	Decreasing γδ T cells in the jejunal epithelium	[123]
FMT with *Clostridium butyricum* and *Saccharomyces boulardii*	Newborn piglets	1.0 × 10^9^ CFU/mL *C. butyricum* and 1.0 × 10^9^ CFU/mL *S. boulardii* were added into the fecal suspension for 3 d	Rise the protein concentrations of IL-23, IL-17A, and IL-22 in the serum	[124]
*Lactobacillus rhamnosus GG*	*S.infantis*-infected weaning piglets	Orally administered 10^10^ CFU for 7 d	Promote the protein level of IL-22 in the serum Enhance protein expression of IL-22 and STAT3 in the ileum	[125]
*Bacillus licheniformis* and *Bacillus subtilis spore* mixture	ETEC-infected weaning piglets	Orally administered 7.8 × 10^8^ CFU for 7 d	Upregulate the mRNA expression of *IL-22* in the small intestine	[126]
*Lactobacillus plantarum*	ETEC-infected suckling Piglets	Orally administered 5 × 10^10^ CFU/Kg BW for 15 d	Decrease the percentage of γδ T cells in the MLNs	[64]
*Lactobacillus reuteri* LR1	Weaning piglets	5 × 10^10^ CFU/Kg diet for 14 d	Increase the protein level of IL-22 in the ileum	[127]
*Lactobacillus rhamnosus* ATCC 7469	ETEC-infected weaning piglets	Orally administrated 10^9^ or 10^11^ CFU for 7 d	Increase serum concentrations of IL-17A Increase the percentage of Th cells in LPLs of small intestine Downregulate mRNA expression of IL-17A in the ileum	[62,128]
*Lactobacillus acidophilus* and *Bacillus subtilis* (FAM®)	Weaning pigs	Basal diet supplemented with 0.1% FAM for 30 d	Increased CD4^+^ T cells in the colonic mucosa Upregulated the expression of IL-22 in the colonic mucosa	[129]
**Fermented feed**				
Fermented Mulberry Leaves	Finishing pigs	10% in diet for 69 d	Increase the contents of IL-22 in plasma	[130]
*Lactobacillus plantarum* GBL fermented feed	Finishing pigs	0.3% RC-LAB fermented feed + probiotics for 75 d	Decrease the mRNA expression of *RORC* and *IL-17A* in MLNs	[131]
co-fermented defatted rice bran	Finishing pigs	Basal diet supplemented with 10% for 30 d	Increase protein levels of IL-22 and IL-23 in the serum	[132]
**Carbohydrate**				
Seaweed-derived polysaccharides and GOS	*Salmonella*-infected finishing pig	Basal diet containing 180 mg laminarin/kg diet and 340 mg fucoidan/kg diet) for 17 d	Reduce mRNA expression level of *IL-22*	[133]
GOS	*Salmonella*-infected finishing pig	2.5 g/kg diet for 17 d	Reduce mRNA expression level of *IL-22*	[133]
Laminarin	*Salmonella*-infected weaning pig	1 g LAM/d in basal diet for 20 d in sows 300 ppm LAM/t diet for 28 d	Reduce mRNA expression of *IL-22* in the colon	[134]
Pectin	LPS-challenged weaning piglets	5% of basal diet for 28 d	Elevate the mRNA expression of *IL-22* in the caecum	[135]
Pectin	Weaning piglets	5% of basal diet for 28 d	Increase the protein level of IL-22 in the serum	[136]
Gouqi polysaccharide	Finishing pigs	0.1% of basal diet for 90 d	Change IL-17 signalling pathways in the jejunum	[137]
Laminarin	Weaning piglets	300 mg/kg basal diet for 8 d	Decrease the mRNA expression of *IL-17A* in the colon	[138]
A combination GMF of GOS, MFGM, and FOS	Suckling piglets	Orally administered 1.2 g/kg BW for 7 d	Upregulate the mRNA expressions of *IL-22* in the ileum	[139]
Chito-oligosaccharides	ETEC-infected weaning piglets	500 mg/kg diet for 10 d	Promote the mRNA expression of *RORC* in the ileum	[140]
Fenugreek seed	Weaning piglets	1.5 g/kg basal diet for 28 d	Increase the relative concentration of the γδ T-cell in the blood	[141]
High dietary fibre	Weaning piglets	6.86% crude fibre for 28 d	Decrease the protein level of IL-17A in the plasma and ileum	[142]
β-glucans	Finishing piglets	0.25 g/kg basal diet for 12 d	Decrease the mRNA expression of *IL-17A*, *IL-17F*, and *IL-22*	[143]
**Nucleotie**				
Yeast-based nucleotide	Suckling piglets	4 g/kg diet from 85 d of gestation to 20 d of lactation	Increase the mRNA expression of *IL-17A* in the jejunum and ileum	[144]
PQQ	ETEC-infected weaning piglets	3 mg/kg basal diet for 14 d	Increase the protein level of IL-22 in the jejunum	[145]
**Globule membrane**				
MFGM	Suckling piglets	1 g/kg BW for 7 d	Increase the mRNA expression of *IL-22* in the ileum and colon	[146]
MFGM	Suckling piglets	9.9 g per day from 85 d of gestation to farrowing	Upregulate the mRNA expression of *IL-22* in the jejunum	[147]
**Energy metabolites**				
AKG	LPS-challenged weaning piglets	1% of basal diet for 21 d	Decrease mRNA expression of *IL-17* and *RORC* in the ileum	[148]
**Immunoglobulins**				
Chicken egg yolk immunoglobulins	ETEC-infected weaning piglets	400 mg/kg basal diet for 6 d	Decrease mRNA expression of *IL-22* in the jejunum and ileum	[149]
**Plants extracts**				
Cathelicidin-WA	Weaning piglets	Intraperitoneal injection with 0.6 mg/kg BW CWA	Decrease the protein level of IL-22 in the jejunum	[150]
Pomelo peel powder	Weaning piglets	8 g PPP/kg diet for 28 d	Decrease the protein level of IL-17A in the serum	[151]
**Enzyme**				
Carbohydrases	Weaning piglets	0.01% of basal diet for 28 d	Increase the mRNA level of *IL-17A* in the colon	[152]
Carbohydrases	Weaning piglets	0.01% of basal diet for 28 d	Reduce the mRNA expression of *IL-22* in the ileum	[153]

## Amino acid

Amino acids play a crucial role in the regulation of immune cells as one of the essential nutrients in mammals. Dietary supplements of gamma-aminobutyric acid (GABA) reduced the feed conversion ratio and protected against ETEC infection via enhanced intestinal immunity, especially the IL-17 response [110,154]. Supplementation of 1% glutamate [112] or glutamine [113] in the basal diet improved production performance and affects the type 3 immune response in lipopolysaccharide (LPS)-challenged piglets. Furthermore, the metabolism of glutamine was essential for the expression of IL-17A in γδ T cells during inflammation in the skin [155]. Besides, the supplementation of putrescine, a key polyamine, or its ornithine, a substrate for polyamine biosynthesis, enhanced IL-22 secretion by ILC3 by enhancing the transcription of IL-22 via nuclear receptor subfamily 4 group A member 1(NR4A1) in mice [156].

## Peptides

Peptides are short chains of amino acids, which are composed of less than 50 amino acids. Peptides are usually derived from the enzymolysis of proteins. It has been demonstrated that hen egg lysozyme [117], soy-derived di- and tripeptides [118], sodium caseinate hydrolysate [119], casein hydrolysate [120], Cathelicidin-BF [157] can all alleviate the expression of type 3 immunity cytokines. These peptides exert the anti-microbial or anti-oxidant function in the gut and then affect the type 3 immune response in cluding the secretion of IL-17A and IL-22, but do not directly regulate the type 3 immune cells. However, it was reported that peptides can regulate the immune cells through cell signals directly in mice. For instance, the Gly-Pro-Ala peptide isolated from fish skin gelatin hydrolysate can significantly regulate the IECs and macrophages via NR4A1 as a ligand [158,159]. Moreover, recent research demonstrated that NR4A1 regulated the ILC3 expansion, and subsequently enhanced gut barrier function and pathogen bacteria defence via IL-22 [160]. In addition, vasoactive intestinal peptides enhanced the resistance of mice to *Citrobacter rodentium* (*C. rodentium*) infection by promoting the C-C Motif Chemokine Receptor 9 (CCR9) expression and intestinal recruitment of ILC3 [161].

## Carbohydrate

Intestinal microbiota extensively participates in the regulation of immune cells. The carbohydrate offers a substrate for the fermentation of intestinal microbiota and then changes the composition and function of microbiota.

In suckling pigs, early life intervention by a combination of galactooligosaccharides (GOS), milk fat globule membrane (MFGM), and fructooligosaccharides (FOS) maintained the intestinal microbial homoeostasis and upregulated the mRNA expressions of *IL-22* in the intestine [139]. Further, pectin or β-glucans increased the mRNA expressions of type 3 immune cytokines *IL-22* and *IL-17* in the intestine in the weaning piglets [119,136]. The underlying mechanisms may be related to the tryptophan metabolites and the AHR signal [136,162]. Tryptophan metabolites, as an energy source, are mainly produced by *lactobacillus* in the intestine and act as ligands for the AHR [163]. Moreover, other microbiota-derived metabolites such as short fatty acids also regulate type 3 immunity. As a substrate for fermentation, fibre such as pectin, GOS, FOS, and β-glucans can be metabolized into diverse short chain fatty acids (SCFAs) in the gut of pig [135,139,143]. The dietary fibre metabolites supported the optimal expansion of ILCs in the intestines through their G-protein-coupled receptors (GPCRs) [164]. In mice, butyrate promoted IL-22 production by CD4^+^ T cells and ILC3 by facilitating hypoxia-inducible factor 1α binding to the hypoxia-response element region of the IL-22 promoter and inducing histone acetylation, thereby enhancing IL-22 expression [165]. Acetate can also promote the secretion of IL-22 in ILC3 through free fatty acid receptor 2 (FFAR2), a fatty acid receptor [166], FFAR2 deletion of ILC3 inhibited ILC3 proliferation in vivo and reduced IL-22 production [167]. Whereas, another type of fatty acid, propionate, directly acts on γδ T cells and inhibits the production of IL-17 and IL-22 in a histone deacetylase-dependent manner [168].

## Trace elements

Iron metabolism is crucial for the fate and function of immune cells in mammalians [169]. In newborn pigs, supplementation of 25 mg d^−1^ Fe in the early life of pigs significantly upregulated protein expression of IL-22 in the colon [114]. A recent study in mice demonstrated that iron deficiency or transferrin receptor (Tfrc) depletion in ILC3 downregulates the AHR signal, and then weakens the function of ILC3, such as the secretion of cytokines IL-17A and IL-22, thus weakening host protection for *C. rodentium* infection [170]. Iron homoeostasis changed under infection, siderophores were increased after ETEC K88 infection in the intestine and siderophore-related gene expression was upregulated in iron deficiency [63]. In mice, the number of IL-22^+^ILC3 and IL-22^+^T cells was reduced in the *Tfrc*^f/f^ *Rorc-cre* mice under *C. rodentium* infection [170]. Furthermore, IL-22 not only took part in the repair of epithelium but also inhibited the growth of pathogenic bacteria by limiting the availability of iron [171]. The above evidence implies that iron metabolism broadly takes part in the regulation of type 3 immunity in the intestines of mammalians. However, the mechanisms by which iron metabolism influences type 3 immune cells in pigs still require further investigation.

A supplementation of nano-ZnO during weaning significantly elevated the numbers of RORγt^+^ T cells in the MLNs in the piglet [115]. In colitis mice, zinc deficiency activates Th17 and aggravates colonic inflammation via the macrophage [172]. There is not enough evidence to show that zinc metabolism directly regulates type 3 immunity now, whereas AHR utilized cellular zinc signals to promote the intestinal barrier function in the epithelium [173]. We speculate zinc may regulate type 3 immunity because RORγt^+^ cells are high in expressing AHR.

Recently, research demonstrated that selenium deficiency increased the secretion of the expression levels of the IL-17A in the intestine of the weaning piglets [116]. Supplementation of selenium can alleviate intestinal inflammation in mice, whereas the function of suppression of Th1 differentiation of selenium serves a vital role [174].

## Probiotics

Directly supplementing probiotics represents a strong strategy for the regulation of type 3 immunity. Treatment of *L. rhamnosus* reduced the expression of IL-22 in the DON-treated jejunal explants of pig [175]. Fecal microbiota transplant (FMT) combined with *C. butyricum* and *S. boulardii* in the early life of pigs significantly upregulated the plasma concentrations of IL-23, IL-17, and IL-22, and also enhanced the intestinal barrier function of piglets after weaning [124]. Further, a lot of research demonstrated that the administration of *Lactobacillus* enhanced the function of type 3 immunity including the number of immune cells [62,129] and production of cytokines [125,127]. The intestinal microbiota-derived GABA also enhanced the IL-17 response. During ETEC infection, *Lactococcus lactis subsp. Lactis-derived* GABA enhanced intestinal IL-17 expression [61]. Mechanistically, GABA promoted Th17 cell responses by activating the GABA- (mammalian target of rapamycin) mTOR signalling pathway [176]. Secondly, indole acted as a ligand for the AHR and can be produced by *Lactobacillus* through tryptophan metabolites and thus enhanced the transcription of IL-22 in ILC3 [162,163,177,178]. Under the states of colorectal cancer, *L. reuteri*-derived indole-3-lactic acid (ILA) inhibited Th17 cell differentiation by targeting the RORγt and suppressed colorectal tumorigenesis [179]. Thirdly, research reported that *L. acidophilus* activates the production of IL-17 and IL-22 of ILC3 and then improves colitis in a nucleotide binding oligomerization domain containing 2 (NOD2)-dependent manner [180]. Whereas it has been demonstrated that NOD2 has high expression in the IECs and myeloid cells, and NOD2 in the myeloid cells took part in the crosstalk between the *E. faecium* and *L. acidophilus* and IL-22^+^ILC3 and IL-22^+^Th cells by SagA [181]. Furthermore, *Lactobacillus* can improve intestinal mucosal formation through the production of L-ornithine from arginine. This, in turn, increases the levels of the AHR ligand L-kynurenine produced from tryptophan metabolism in IECs, ultimately leading to an increase in IL-22^+^ILC3 [182].

Further, *L. plantarum* GBL fermented feed decreased the mRNA expression of transcription factors RORγt and cytokines in Th17 [131]. However, since we do not yet know which specific substance primarily regulates this function, further exploration to identify more refined mechanisms is warranted.

## Others

MFGM is a triple-layer structure that surrounds the fat globules in milk, which are composed of proteins, lipids, and carbohydrates. Administered MFGM directly in the suckling pigs or supplemented with MFGM in the sow basal diet during suckling significantly increased the *IL-22* and *Reg3γ* expression in ileal and colonic mucosa [146,147]. Besides, previous studies showed that plant extracts such as amidoamine or proanthocyanins [183], carbohydrases [152], Yeast-based nucleotides [144], and Alpha-ketoglutarate (AKG) [148] also regulate the type 3 immune response in the pig.

Besides, there are some nutrients have been confirmed in mice to regulate the development of type 3 immune cells, but further confirmation is needed in pigs. Vitamins are crucial nutrients that play a vital role in maintaining normal physiological functions. Their deficiency increased the susceptibility and severity of infections caused by pathogens. Vitamin D and A are required for the function of ILC3 cells and protection from *C. rodentium* infection [184,185]. Further, retinoic acid, a vitamin A metabolite, increased IL-22 secretion by γδ T cells and ILC3 under *C. rodentium* infection [186].

## Conclusion and future perspectives

In summary, type 3 immunity has attracted much attention in recent years for its broad response to infection, inflammation, and stress. The usual diarrhoea-relative pathogens such as ETEC and PEDV cause a type 3 immune response. However, the immature intestinal immune system of neonatal and weaning piglets renders them inefficient immune responses to pathogens compared with the finishing stage. This is the primary factor that contributes to the occurrence of gastrointestinal diseases. Accelerating the development of type 3 immunity by nutritional interventions may be a strong strategy to prevent the occurrence of gastrointestinal diseases and promote growth performance in pigs.

We found studies on type 3 immunity stay on the level of gene and protein in the intestinal tissue of pigs. Much frontier technology including FISH, multiplex immunofluorescence, flow cytometry, and scRNA-seq should be applied in the pig model to research the immune cells themselves. Given the difficulties of living model testing, we advocate more vitro models, for example, LPLs incubating [170] and intestinal explants incubating [187], which makes it easy to evaluate the effects of various nutrients or pathogens on type 3 immunity. While it is acceptable to evaluate responses via in vitro models, it is critical to validate these responses in vivo later. Further, a recent study demonstrated that the microbiome contributes to the function of type 3 immune response by using the germ-free pig model, especially the IL-17-producing [15]. This guides us to investigate many efficient tools for regulating type 3 immune cells via microbiota strategies, such as probiotics, prebiotics, and postbiotics.

Regarding type 3 immunity in humans, current research remains incomplete. While studies on human γδ T cells have provided some insights [16], other type 3 immune cells are less well understood. This may be attributed to challenges in sample collection and the need for advanced technologies such as single-cell omics and spatial omics to further elucidate the dynamics of type 3 immunity in humans. Further research is necessary to bridge these gaps and enhance our understanding of type 3 immunity in the human context.

Because of the similarities in genome, anatomy, and physiology between pigs and humans, the pig model seems to be better suitable for the research of the interaction of microbiome and type 3 immunity in humans and thus solve the intestinal health of babies. However, while the pig model offers valuable insights, it is not without its limitations. First, there are discrepancies in cell populations, such as γδ T cells, which can affect the generalizability of findings. Second, the technology for gene-edited pigs is still under development, and the lack of sufficient gene knockout models poses challenges for in-depth mechanistic studies. These limitations highlight the need for continued refinement of the pig model to fully understand and leverage its potential in immunological research.

## Abbreviations

IL: interleukin
RORγt: retinoic acid-related orphan receptor
ILCs: innate lymphoid cells
γδ T: gamma delta T cell
Th: T helper
TCR: T cell receptor
IELs: intraepithelial lymphocytes
PBMC: peripheral blood mononuclear cells
MLNs: mesenteric lymph nodes
T-bet: T-box transcription factor 21
IFN: interferon
GATA3: GATA binding protein 3
scRNA-seq: Single-cell RNA Sequencing
LPLs: lamina propria lymphocytes
AHR: aryl hydrocarbon receptor
FISH: fluorescence in situ hybridization
Reg3γ: regenerating family member 3 gamma
CXCL-13: C-X-C motif chemokine ligand 13
MUC17: Mucine17
IECs: intestinal epithelial cells
DON: deoxynivalenol
ETEC: Enterotoxigenic Escherichia coli
IPEC-J2: intestinal porcine epithelial cell line J2
PEDV: porcine epidemic diarrhoea virus
*B.hyodysenteriae*: *Brachyspira hyodysenteriae*
*C.perfringens*: *Clostridium perfringens*
*S*. Typhimurium: *Salmonella* Typhimurium
NIV: Nivalenol
GABA: gamma-aminobutyric acid
LPS: lipopolysaccharide
GPA: Gly-Pro-Ala
*C. rodentium*: *Citrobacter rodentium*
CCR9: C-C Motif Chemokine Receptor 9
GOS: galactooligosaccharides
MFGM: milk fat globule membrane
FOS: fructooligosaccharides
FFAR2: free fatty acid receptor 2
SCFAs: short chain fatty acids
Tfrc: transferrin receptor
FMT: faecal microbiota transplant
ILA: indole-3-lactic acid
mTOR: mammalian target of rapamycin
NOD2: nucleotide binding oligomerization domain containing 2
AKG: Alpha-ketoglutarate
PQQ: Pyrroloquinoline quinone

## Data Availability

Data sharing is not applicable to this article as no new data were created or analysed in this study.
